# *WRR4B* contributes to a broad‐spectrum disease resistance against powdery mildew in *Arabidopsis*

**DOI:** 10.1111/mpp.13415

**Published:** 2024-01-08

**Authors:** Shuangshuang Mei, Yuxin Song, Zuer Zhang, Haitao Cui, Shuguo Hou, Weiguo Miao, Wei Rong

**Affiliations:** ^1^ College of Plant Protection Hainan University Haikou Hainan China; ^2^ Key Laboratory of Green Prevention and Control of Tropical Plant Diseases and Pests Hainan University, Ministry of Education Haikou Hainan China; ^3^ Shandong Provincial Key Laboratory of Agricultural Microbiology, College of Plant Protection Shandong Agricultural University Tai'an Shandong China; ^4^ Institute of Advanced Agricultural Sciences Peking University Weifang Shandong China

**Keywords:** *Arabidopsis*, broad‐spectrum resistance, powdery mildew, *WHITE RUST RESISTANCE 4B*

## Abstract

*Oidium heveae* HN1106, a powdery mildew (PM) that infects rubber trees, has been found to trigger disease resistance in *Arabidopsis thaliana* through ENHANCED DISEASE SUSCEPTIBILITY 1 (EDS1)‐, PHYTOALEXIN DEFICIENT 4 (PAD4)‐ and salicylic acid (SA)‐mediated signalling pathways. In this study, a typical *TOLL‐INTERLEUKIN 1 RECEPTOR, NUCLEOTIDE‐BINDING, LEUCINE‐RICH REPEAT* (*TIR‐NB‐LRR*)‐encoding gene, *WHITE RUST RESISTANCE 4* (*WRR4B*), was identified to be required for the resistance against *O. heveae* in *Arabidopsis*. The expression of *WRR4B* was upregulated by *O. heveae* inoculation, and *WRR4B* positively regulated the expression of genes involved in SA biosynthesis, such as *EDS1*, *PAD4*, *ICS1* (*ISOCHORISMATE SYNTHASE 1*), *SARD1* (*SYSTEMIC‐ACQUIRED RESISTANCE DEFICIENT 1*) and *CBP60g* (*CALMODULIN‐BINDING PROTEIN 60 G*). Furthermore, WRR4B triggered self‐amplification, suggesting that WRR4B mediated plant resistance through taking part in the SA‐based positive feedback loop. In addition, WRR4B induced an *EDS1*‐dependent hypersensitive response in *Nicotiana benthamiana* and contributed to disease resistance against three other PM species: *Podosphaera xanthii*, *Erysiphe quercicola* and *Erysiphe neolycopersici*, indicating that *WRR4B* is a broad‐spectrum disease resistance gene against PMs.

## Graphical abstract

*WRR4B* mediates broad‐spectrum disease resistance against powdery mildews *Oidium heveae*, *Podosphaera xanthii*, *Erysiphe quercicola* and *E. neolycopersici* through taking part in a salicylic acid‐based positive feedback loop.

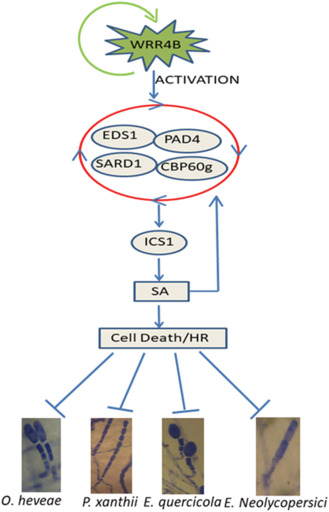

## 1 INTRODUCTION

Powdery mildew (PM) fungi are obligate biotrophic phytopathogens that need living plant host cells for development and propagation. More than 650 PM species have been reported to colonize nearly 10,000 plant species, including crop and ornamental plants (Glawe, 2008; Takamatsu et al., 2008), causing significant agricultural and economic losses worldwide (Attanayake et al., 2009; Dean et al., 2012; Glawe, 2008; Linde et al., 2006). *Oidium heveae*, the causal agent of PM disease on rubber trees (*Hevea brasiliensis*), can infect young host tissues and greatly reduce rubber yields (Saranya et al., 2005). Upon encountering a host cell, PM fungi develop an appressorium to penetrate the cell wall to form a feeding structure, the haustorium, that continuously takes nutrients from the plant cell until mature spore offspring are produced (Mei et al., 2016). The typical white powdery appearance represents a combination of fungal mycelium and asexual propagation structures (conidiophores and conidia), which become apparent on the surface of aboveground plant organs at later stages of infection.

PM fungi secrete a large number of effector proteins into plant cells to manipulate the physiological processes of its host to facilitate its invasion (Thordal‐Christensen et al., 2018). Correspondingly, some plants have evolved dominantly or semi‐dominantly inherited resistance (*R*) genes that detect pathogen effectors inside the plant cell to trigger a hypersensitive response (HR) associated with local host cell death to limit the spread of the fungus (Bourras et al., 2015; Jones & Dangl, 2006; Lu et al., 2016; Praz et al., 2017). These *R* genes typically encode canonical nucleotide‐binding leucine‐rich repeat (NB‐LRR/NLR) proteins and represent the most variable gene family in plants (Jacob et al., 2013; Jones et al., 2016). Whole‐genome sequencing has indicated that higher plant species possess anywhere from 50 (papaya, *Carica papaya*) to over 1500 (wheat, *Triticium aestivum*) *NLR* genes (Gao et al., 2018; Porter et al., 2009), with more than 150 *NLR* genes in *Arabidopsis thaliana* (Meyers et al., 2003). The expression levels of *NLR*s are strictly regulated (Lai & Eulgem, 2018), with low levels observed in healthy plants and highly variable levels in plants challenged by particular pathogens (van Wersch et al., 2020). The NLR protein must reach a certain abundance (dose) to activate defence signalling, but the overexpression of *NLR*s often results in autoimmunity, with massive fitness costs (Bieri et al., 2004; Holt et al., 2005; Lai & Eulgem, 2018). The N‐terminal coiled‐coil (CC) or Toll/interleukin‐1 receptor (TIR) domains are used to classify plant NLRs into two main groups, termed CC‐NLRs (CNLs) and TIR‐NLRs (TNLs) (Meyers et al., 2003; Monteiro & Nishimura, 2018; van Wersch et al., 2020). Both the CC and TIR domains play key roles in the formation of oligomers that are essential for the activation of NLRs and the transduction of downstream immune responses (Bernoux et al., 2011; Maekawa, Cheng, et al., 2011). Generally, CNLs are present in monocot and dicot plants, whereas TNLs are only represented in dicots (Jacob et al., 2013).

Several key components involved in effector‐triggered immunity (ETI) signalling were isolated through different genetic screens. The first identified gene involved in ETI signalling was *NON‐RACE‐SPECIFIC DISEASE RESISTANCE 1* (*NDR1*), which was found to be necessary for resistance against bacterial and fungal pathogens carrying a variety of effectors (Century et al., 1995). The loss of another gene, *ENHANCED DISEASE SUSCEPTIBILITY 1* (*EDS1*), disabled the immunity conferred by several NLRs in the RESISTANCE TO *Peronospora parasitica* (*RPP*) family (Parker et al., 1996). In general, NDR1 transduces CNL signals and EDS1 transduces TNL signals, suggesting that there are at least two distinct downstream signalling branches upon NLR activation (Aarts et al., 1998). Further studies revealed that TNLs have NADase activity and that the AMP, ADP and ATP‐related products specifically activate the EDS1–PAD4 (PHYTOALEXIN DEFICIENT 4) or EDS1–SAG101 (SENESCENCE‐ASSOCIATED GENE 101) heteromeric complexes to regulate TNL‐triggered immunity (Huang et al., 2022; Jia et al., 2022).

Salicylic acid (SA)‐dependent local and systemic resistance pathways play pivotal roles in the defence against biotrophic and hemibiotrophic pathogens (Chandran et al., 2009; Zimmerli et al., 2004). A hallmark of ETI signalling is the strong and rapid transcriptionally mobilized resistance controlled by SA, embodied in the transcript accumulation of SA‐responsive transcription factors, the SA biosynthesis gene *ISOCHORISMATE SYNTHASE 1* (*ICS1*) and SA‐responsive pathogenesis‐related (*PR*) genes such as *PR1* (Seyfferth & Tsuda, 2014; Zhang & Li, 2019). In TNL immunity, EDS1 and its partner PAD4 function upstream of SA signalling to positively regulate *ICS1* expression and SA accumulation (Falk et al., 1999; Feys et al., 2001; Wagner et al., 2013; Zhou et al., 1998). Furthermore, a positive feedback loop exists in which elevated SA levels promote the expression of *EDS1*, *PAD4* and other genes, for example, *SYSTEMIC‐ACQUIRED RESISTANCE DEFICIENT 1* (*SARD1*) and *CALMODULIN‐BINDING PROTEIN 60 G* (*CBP60g*) to amplify the resistance outputs (Cui et al., 2017; Ngou et al., 2022; Zeier, 2021). In addition, the feedback loop also upregulates the expression of pipecolic acid (*N*‐hydroxyl‐pipecolic acid [NHP])‐biosynthesis genes, such as *FLAVIN‐CONTAINING MONOOXYGENASE 1* (Liu et al., 2020), enabling mutual reinforcement and protection against pathogen interference.

Our previous studies have shown that the PM species *O. heveae* HN1106 triggers the HR in *Arabidopsis* through EDS1 and PAD4 but not on NDR1, suggesting that TNL‐triggered immunity is probably involved in the resistance against *O. heveae* (Mei et al., 2016). To date, however, none of the canonical *NLR* genes have been identified to confer resistance to PMs in *Arabidopsis*. In this study, one typical TNL gene, *WHITE RUST RESISTANCE 4* (*WRR4B*), was found to be involved in the disease resistance against *O. heveae*. The expression of *WRR4B* was upregulated, and *WRR4B* positively controlled the *EDS1*, *PAD4* and SA pathways during *O. heveae* infection. Furthermore, overexpressing *WRR4B* induced cell death in *Nicotiana benthamiana* in an *NbEDS1*‐dependent manner. These findings lay the foundation for further study of the molecular mechanisms by which *WRR4B* protects against PMs.

## 2 RESULTS

### 2.1 *WRR4B* contributes to the resistance against *O. heveae* HN1106

**FIGURE 1:**
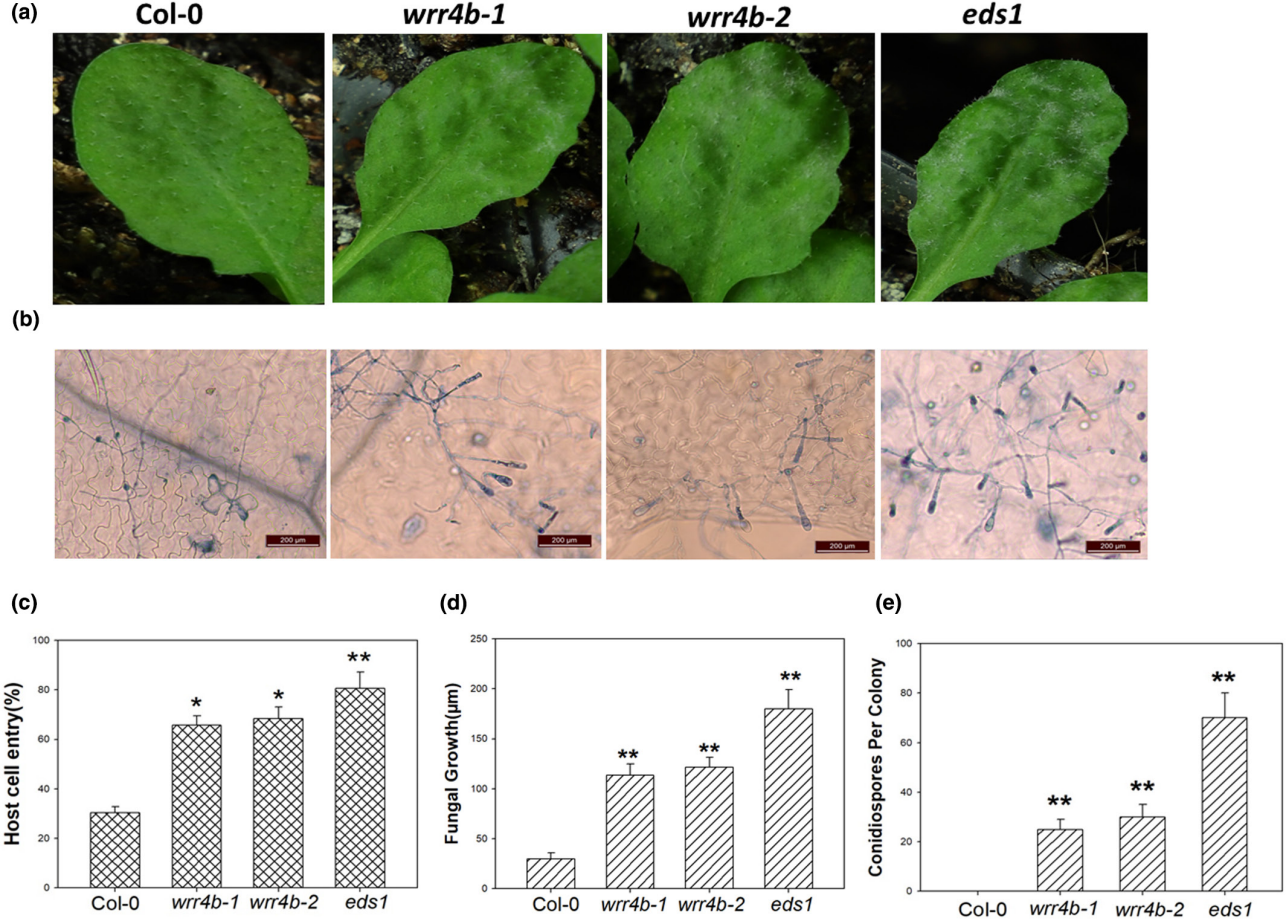
*WRR4B* contributes to the resistance against *Oidium heveae* HN1106. Five‐week‐old plants were inoculated with *O. heveae* HN1106. (a) Leaf symptoms at 6 days post‐inoculation (dpi). (b) Fungal structures stained with trypan blue and imaged using a light microscope at 6 dpi. Bars, 200 μm. (c) Quantitative analysis of host cell entry rates at 1 dpi; at least 50 germinated sporelings/leaves were counted for each experiment. (d) Quantitative analysis of *O. heveae* hyphal growth at 2 dpi. (e) The numbers of conidiospores per colony were counted at 6 dpi. Twenty fungal colonies were analysed per genotype for (d) and (e). The data represent the mean ± *SD* of three experiments. Significant difference from Col‐0 by Student's *t*‐test, *p < 0.05, **p < 0.01. These experiments were repeated twice with similar results.

*O. heveae* infection triggers host disease resistance mechanisms dependent on EDS1 and PAD4, but not on NDR1 (Mei et al., 2016), suggesting that TIR‐NBS‐LRR proteins are responsible for *O. heveae*‐induced resistance in *Arabidopsis*. To identify the genes responsible, we performed an RNA‐sequencing (RNA‐seq) analysis of wild‐type (WT) *A. thaliana* Col‐0 plants inoculated with *O. heveae* HN1106 at 0 and 2 days post‐inoculation (dpi) and found that 12 genes encoding TIR‐NBS‐LRR proteins were differentially expressed in the infected plants, with nine being upregulated and three being downregulated (Table S1). To determine whether these genes are involved in the resistance against *O. heveae*, homozygous T‐DNA insertion lines were obtained for each of the *TIR*‐*NBS*‐*LRR* genes (Table S1). These mutants, together with the *Arabidopsis* WT Col‐0 and *eds1* mutants, were inoculated with *O. heveae*. At 6 dpi, no obvious changes were observed on the Col‐0 leaves. In contrast, the leaf surface of one mutant (SALK_040895C, *wrr4b‐1*) displayed mild white cotton‐like symptoms (Figure 1a), but the other 11 T‐DNA insertion lines appeared to be unaffected (Table S2). The *eds1* mutant developed large and coalesced white patches over the entire leaf surface (Figure 1a). Next, we examined the fungal growth of *O. heveae* on the WT and mutants. Trypan blue staining showed that *O. heveae* formed dense hyphal networks and many conidiospores on *wrr4b‐1* and *eds1* (Figure 1b,e), but only limited fungal growth and no conidiospore formation was observed on WT (Figure 1b) and the other 11 T‐DNA insertion mutants (Table S2). The SALK_040895C T‐DNA insertion is located within the second exon of *AT1G56540* (*WRR4B*), which was previously reported to be involved in the resistance against the white rust pathogen *Albugo candida* (Cevik et al., 2019). To further confirm the function of *WRR4B* in the resistance against *O. heveae*, another T‐DNA insertion line, SALK_072335C (*wrr4b‐2*), was inoculated with *O. heveae*. By 6 dpi, *wrr4b‐2* also displayed a PM‐susceptible phenotype with white patches on the leaf surface (Figure 1a); however, fewer conidiospores on the *wrr4b‐1* and *wrr4b‐2* mutants than on the *eds1* mutant were formed (Figure 1b,e).

In addition, we tested whether *WRR4B* is required for the early disease resistance during *O. heveae* infections in *Arabidopsis*. Successful host cell penetration and hyphal growth are early events in PM pathogenesis. We calculated the plant cell penetration ratios and measured the length of fungal growth in the early stages of infection. By 1 dpi, there was a host cell entry rate of about 60% on the *wrr4b‐1* and *wrr4b‐2* mutants, in contrast to 37% in Col‐0 and 78% in the *eds1* mutant (Figure 1c). By 2 dpi, the hyphae had elongated to over 100 μm on the *wrr4b‐1* and *wrr4b‐2* mutants, 180 μm on the *eds1* mutant, but only about 35 μm on the WT (Figure 1d). Overall, these results indicated that *WRR4B* contributes to resistance against *O. heveae* in the early infection stage of *Arabidopsis*.

### 2.2 *O. heveae* HN1106 triggers cell death and H_2_O_2_ production on *wrr4b* mutants in the later stage of infection

**FIGURE 2:**
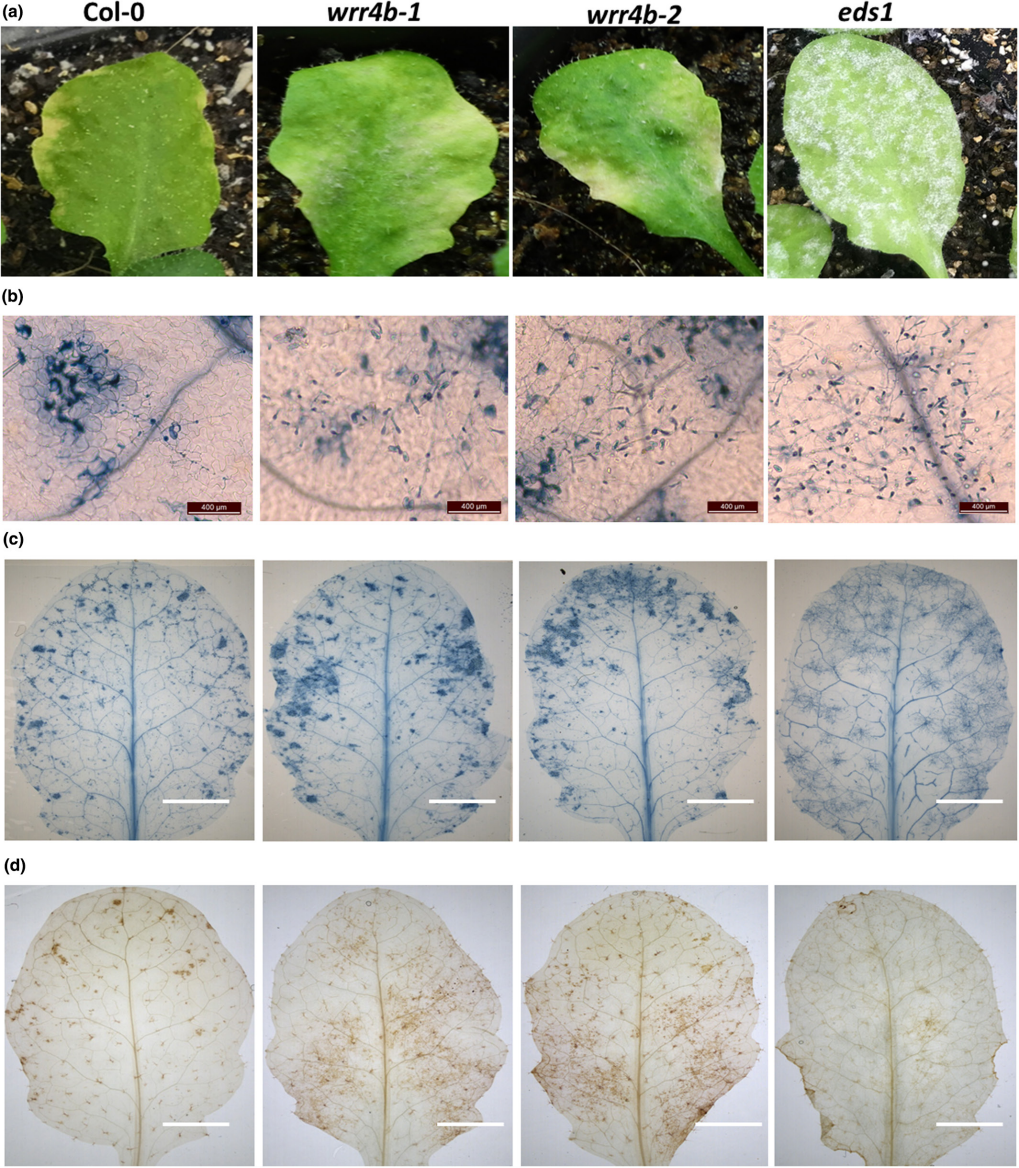
The cell death and H_2_O_2_ production in the later stage of *Oidium heveae* infection. Five‐week‐old plants were inoculated with *O. heveae* HN1106. (a) Leaf symptoms at 12 days post‐inoculation (dpi). (b) Microscopy analysis of infected leaves with trypan blue staining at 12 dpi. Bars, 400 μm. (c) The whole infected leaves were photographed by a light stereoscope with trypan blue staining at 12 dpi. Bars, 5 mm. (d) The whole infected leaves were photographed by a light stereoscope with 3,3′‐diaminobenzidine staining. Bars, 5 mm. These experiments were repeated twice with similar results.

A previous study has shown that *O. heveae* triggers chlorosis symptoms and induces cell death and H_2_O_2_ production on WT Col‐0 (Mei et al., 2016). To determine whether *O. heveae* can also induce these defence responses in *wrr4b* mutants, WT Col‐0, *wrr4b‐1*, *wrr4b‐2* and *eds1* mutants were inoculated with *O. heveae* HN1106. By 12 dpi, chlorosis symptoms occurred on the edge of the WT Col‐0 leaves, and the typical white powder appeared on the whole leaf surfaces of the *eds1* mutant (Figure 2a), which is consistent with the previous report (Mei et al., 2016). Unlike the WT Col‐0 and *eds1* mutants, the *wrr4b‐1* and *wrr4b‐2* mutants developed large necrosis patches with a few white powders across the leaf surfaces (Figure 2a). Microscopy analysis showed that dense hyphal networks, some conidiospores and mature fungal spores were observed on *wrr4b‐1*, *wrr4b‐2* and *eds1* mutants but not on WT Col‐0 and the other 11 *TIR*‐*NB*‐*LRR* T‐DNA insertion lines (Figure 2b, Table S2). Fewer mature fungal spores were present on the *wrr4b‐1* and *wrr4b‐2* tissues than were detected for the *eds1* mutant (Figure 2b). In addition, the patches of cell death and H_2_O_2_ production induced by *O. heveae* on the *wrr4b‐1* and *wrr4b‐2* mutants were both slightly greater than those induced on WT (Figure 2c,d). The *eds1* mutant did not display fungal‐induced cell death and H_2_O_2_ production (Figure 2c,d). These results show that *O. heveae* still triggers apparent cell death and H_2_O_2_ production on the *wrr4b* mutants, as well as on WT *Arabidopsis*.

### 2.3 The expression of *WRR4B* is upregulated by *O. heveae*

**FIGURE 3:**
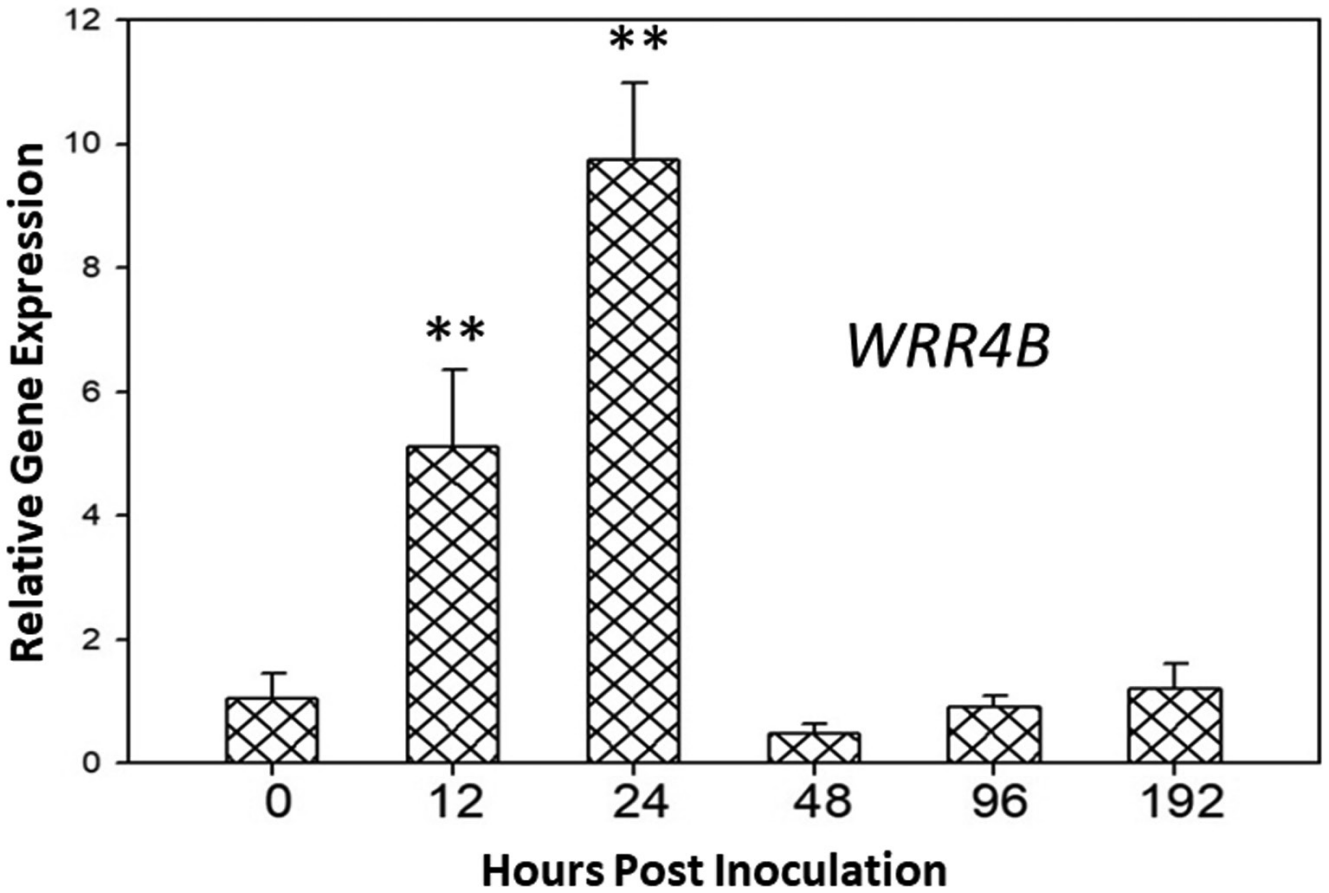
The expression of *WRR4B* is upregulated by *Oidium heveae*. Five‐week‐old *Arabidopsis* wild‐type (WT) Col‐0 was inoculated with *O. heveae* HN1106. The abundance of *WRR4B* mRNA was determined at the indicated time points after *O. heveae* inoculation using reverse transcription‐quantitative PCR. The data represent the mean ± *SD* of three independent experiments and six RNA replicates for each experiment. Significant difference from 0 h post‐inoculation (hpi) of Col‐0 by Student's *t*‐test, **p < 0.01. These experiments were repeated twice with similar results.

Plants display large‐scale changes in the expression levels of cognate *NLR* genes when they are challenged by pathogens, with many *R* genes being upregulated during the infection process (Lai & Eulgem, 2018; Tan et al., 2007; van Wersch et al., 2020). By contrast, in the present study, our RNA‐seq analysis revealed that *WRR4B* was moderately downregulated by *O. heveae* at 2 dpi. To further explore the mechanisms regulating *WRR4B* expression following *O. heveae* infection at varying time points, WT Col‐0 was inoculated with *O. heveae*. Total RNA was isolated from plant samples at 0, 12, 24, 48, 96 and 192 h post‐inoculation (hpi), and reverse transcription‐quantitative PCR (RT‐qPCR) was used to monitor *WRR4B* expression. Compared with its level at 0 dpi, the abundance of *WRR4B* mRNA was significantly enhanced at 12 and 24 hpi (Figure 3); however, the expression of *WRR4B* was reduced at 48 hpi and returned to the initial level of 0 hpi (Figure 3) at 96 and 192 hpi, suggesting that *WRR4B* expression is upregulated in the early stages of *O. heveae* infection.

### 2.4 *WRR4B* positively regulates the expression of genes involved in SA biosynthesis by a self‐amplification loop

**FIGURE 4:**
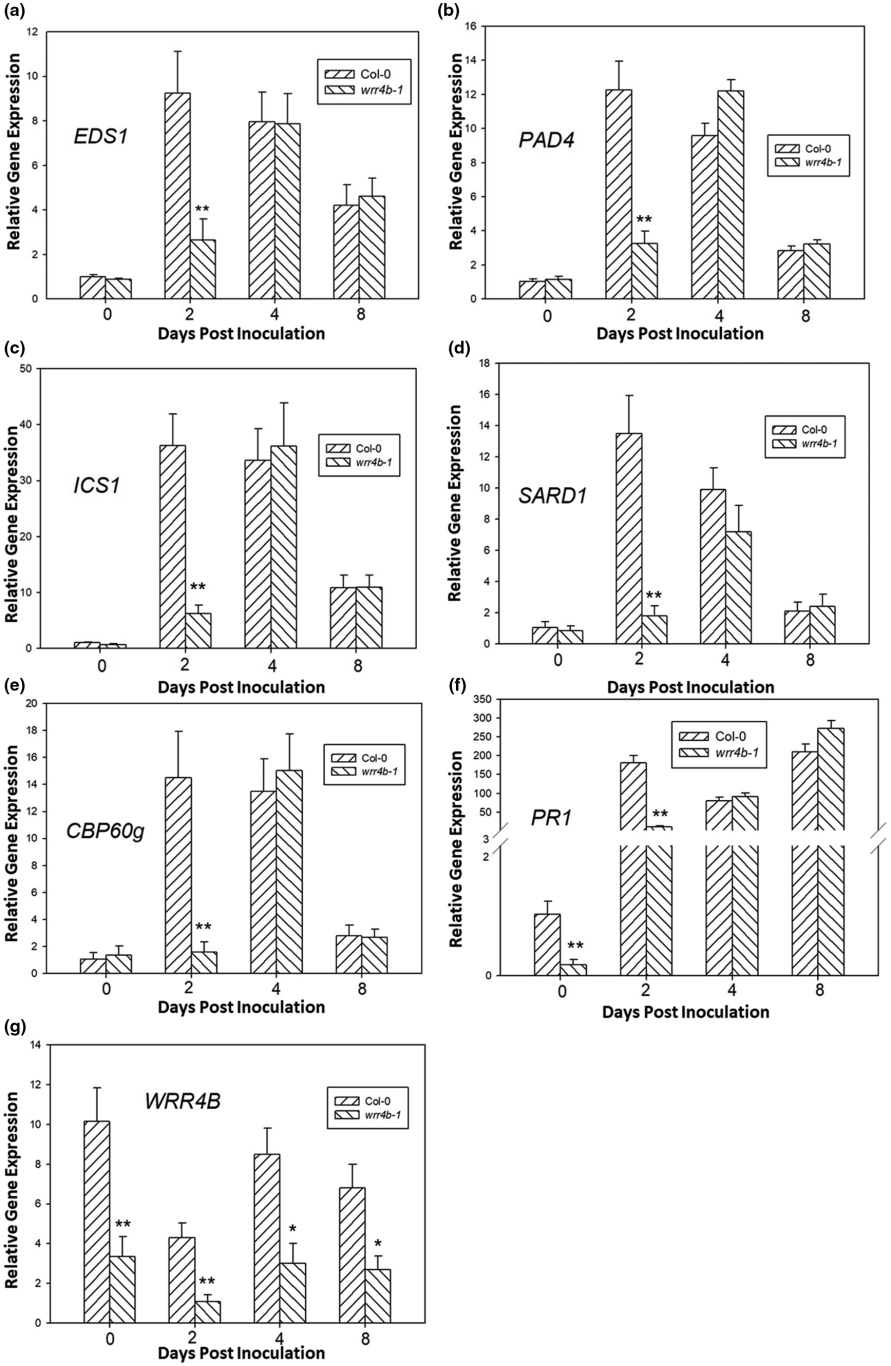
*WRR4B* positively regulates the expression of *EDS1*, *PAD4*, *ICS1*, *SARD1*, *CBP60g*, *PR1* and *WRR4B*. Five‐week‐old *Arabidopsis* wild‐type Col‐0 (WT) and *wrr4b‐1* plants were inoculated with *Oidium heveae* HN1106. The abundances of *EDS1* (a), *PAD4* (b), *ICS1* (c), *SARD1* (d), *CBP60g* (e), *PR1* (f) and *WRR4B* (g) mRNA were determined at the indicated time points after *O. heveae* infection using reverse transcription‐quantitative PCR. The data represent the mean ± *SD* of three independent experiments and six RNA replicates for each experiment. Significant difference from Col‐0 by Student's *t*‐test, *p < 0.05, **p < 0.01. These experiments were repeated twice with similar results.

Activation of TNLs leads to the upregulation of SA biosynthesis‐related genes, such as *EDS1*, *PAD4*, *ICS1*, *SARD1* and *CBP60g* (Ngou et al., 2022; Zeier, 2021), and the EDS1, PAD4 as well as SA pathway are required for *O. heveae*‐triggered resistance in *Arabidopsis* (Mei et al., 2016). In the present study, the TIR‐NB‐LRR‐type gene *WRR4B* was shown to contribute to resistance against *O. heveae*; therefore, we hypothesized that *WRR4B* may function by regulating the expression of genes involved in SA signalling. To test this hypothesis, WT and *wrr4b‐1* mutant plants were inoculated with *O. heveae*. Plant leaves were collected at 0, 2, 4 and 8 dpi, and the transcript levels of *EDS1*, *PAD4*, *ICS1*, *SARD1*, *CBP60g* and *PR1* were determined using RT‐qPCR. Compared with the WT, the expression levels of *EDS1*, *PAD4*, *ICS1*, *SARD1*, *CBP60g* and *PR1* were significantly downregulated in the mutant at 2 dpi (Figure 4a–e). At 0, 4 and 8 dpi, the abundances of *EDS1*, *PAD4*, *SARD1*, *CBP60g* and *ICS1* mRNA in the *wrr4b‐1* mutant were almost the same as those in the WT (Figure 4a–e). The *PR1* mRNA abundance in the *wrr4b‐1* mutant was much lower than in the WT at 0 and 2 dpi, but almost identical to the WT at 4 and 8 dpi (Figure 4f). Compared with their levels at 0 dpi, the expression of *EDS1*, *PAD4, SARD1*, *CBP60g* and *ICS1* was more strongly induced at 2 and 4 dpi and mildly upregulated at 8 dpi (Figure 4a–e), whereas *PR1* expression was strongly induced at 2, 4 and 8 dpi both in WT and mutant (Figure 4f). In addition, the expression of *WRR4B* itself was obviously downregulated in the *wrr4b‐1* mutant at each time point (Figure 4g). Taken together, these results indicate that *WRR4B* triggers self‐amplification and positively regulates the expression of SA biosynthesis‐related genes during the early stage of *O. heveae* infection.

### 2.5 The transient expression of *WRR4B* induces *Nb‐EDS1* dependent hypersensitive responses in *N. benthamiana*

**FIGURE 5:**
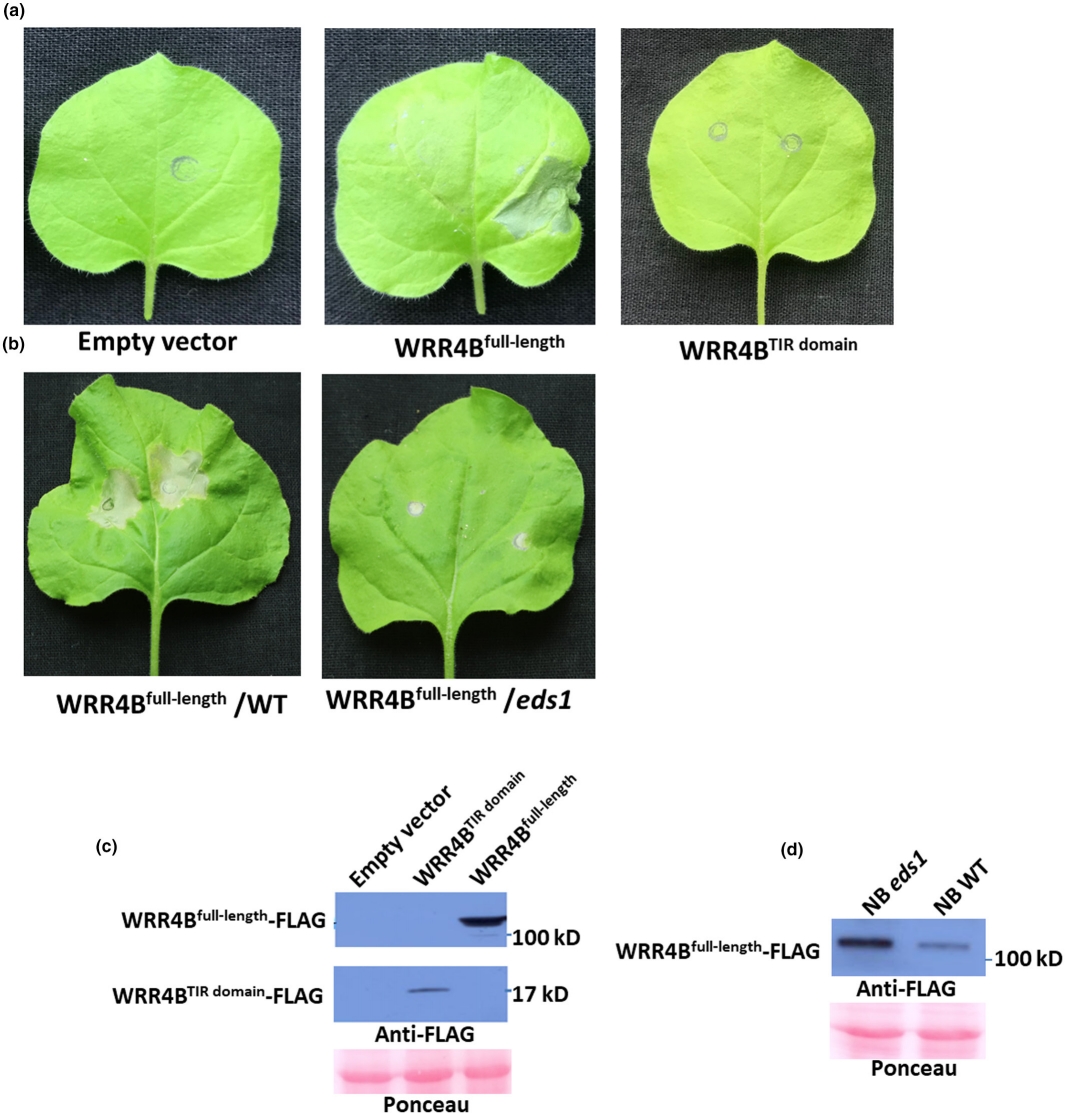
Oestrogen‐inducible *WRR4B* expression triggers a hypersensitive response in *Nicotiana benthamiana*. Five‐week‐old wild‐type (WT) *N. benthamiana* and *Nbeds1* mutant leaves were infiltrated with *Agrobacterium tumefaciens* GV3101, and the plants were sprayed with oestradiol at 1 day post‐inoculation (dpi). Symptoms were photographed at 2 days post‐oestradiol treatment. (a) The symptoms of WT *N. benthamiana* leaves inoculated with *A. tumefaciens* GV3101 carrying the pER8 empty vector, pER8‐*WRR4B* ^full‐length coding sequence^ or pER8‐*WRR4B* ^TIR domain^. (b) The symptoms of WT *N. benthamiana* and *Nbeds1* mutant leaves inoculated with *A. tumefaciens* GV3101 carrying the pER8‐*WRR4B* ^full‐length coding sequence^. (c, d) The protein abundance was determined using an immunoblot assay with an anti‐FLAG antibody at 24 h post‐oestradiol treatment. These experiments were repeated twice with similar results.

Elevated R protein expression levels often induce cell death to inhibit the spread of pathogens (Bieri et al., 2004; Holt et al., 2005; Lai & Eulgem, 2018), and only the TIR domain of some NLRs is sufficient to induce cell death (Bernoux et al., 2011; Maekawa, Kufer, et al., 2011; Wan et al., 2019). To gain further insight into the function of *WRR4B*, the oestrogen‐inducible constructs pER8‐*WRR4B* ^full‐length coding sequence^ and pER8‐*WRR4B* ^TIR domain^ were made. We transiently expressed them in *N. benthamiana* by infiltrating the plants with *Agrobacterium tumefaciens* carrying the indicated vectors, and at 1 dpi, the *N. benthamiana* leaves were sprayed with oestrogen. Only the full‐length *WRR4B* induced typical cell death symptoms in the injection region of the *N. benthamiana* leaves 2 days after the oestrogen treatment (Figure 5a,c). No obvious changes were observed for the *N. benthamiana* leaves infiltrated with *A. tumefaciens* carrying an empty vector or carrying the vector containing the TIR domain of *WRR4B* alone (Figure 5a,c). In addition, we further expressed *WRR4B* ^full‐length^ in WT *N. benthamiana* and *Nb*‐*eds1* mutant leaves. The strong HR was observed on WT *N. benthamiana* but not on the *Nb*‐*eds1* mutant after 2 days of treatment with oestrogen (Figure 5b,d). Overall, these results indicated that transient expression of *WRR4B* induced cell death in *Nb‐EDS1‐*dependent manner in *N. benthamiana*.

### 2.6 *WRR4B* contributes to a broad‐spectrum disease resistance against PMs

**FIGURE 6:**
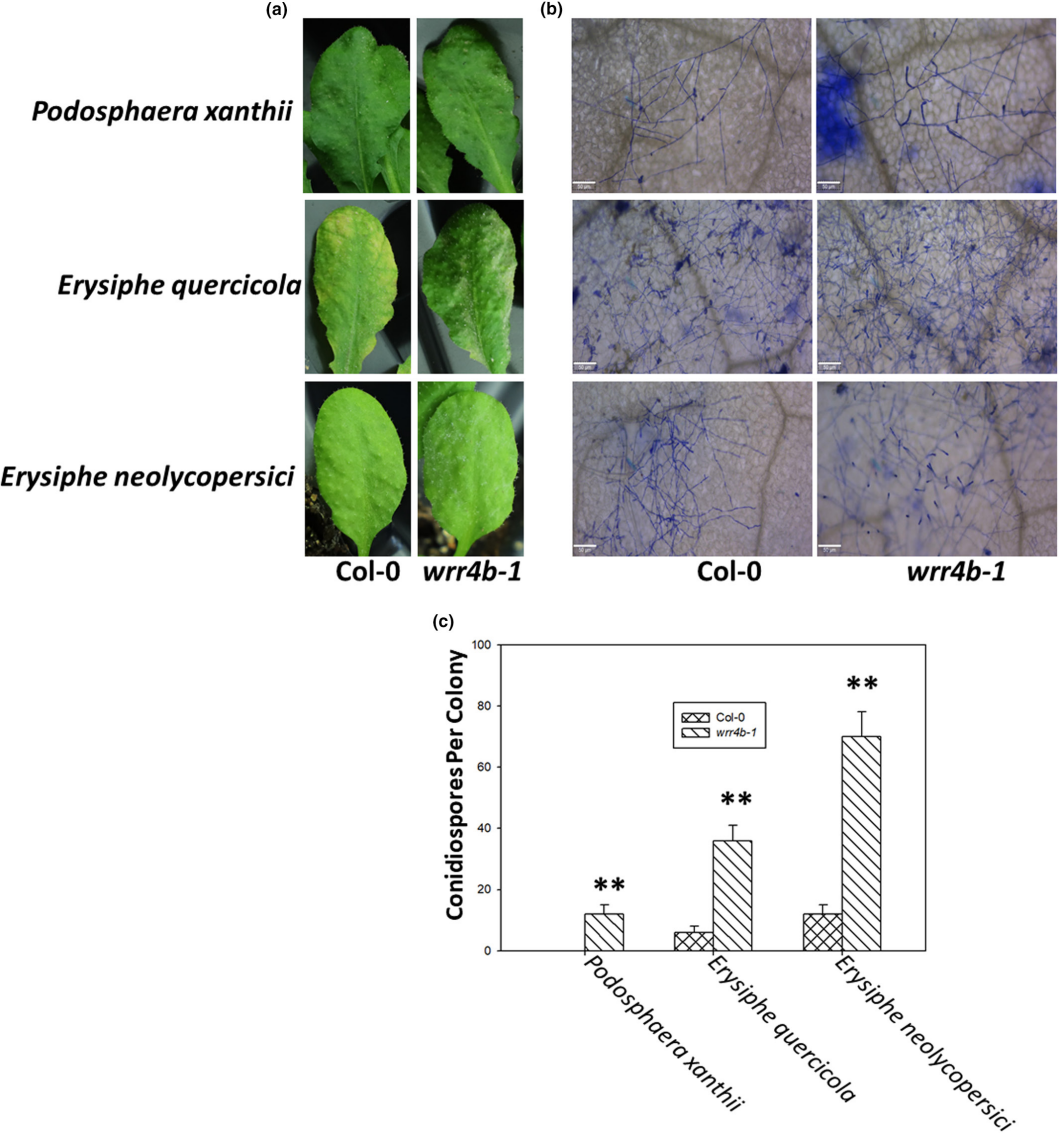
*WRR4B* contributes to resistance against *Podosphaera xanthii*, *Erysiphe quercicola* and *Erysiphe neolycopersici*. Five‐week‐old *Arabidopsis* wild‐type (WT) Col‐0 and *wrr4b‐1* mutants were infected with *P. xanthii* JD 2202, *E. quercicola* JLX 2201 and *E. neolycopersici* FQ 2205. (a) Leaf symptoms. (b) Fungal structures stained with Coomassie blue were imaged using a light microscope. Bars, 50 μm. (c) The numbers of conidiospores per colony were counted. The data represent the mean ± *SD* of at least 20 fungal colonies per genotype. These experiments were repeated twice with similar results. Significant difference from Col‐0 by Student's *t*‐test, **p < 0.01.

*WRR4B* is involved in the disease resistance against *O. heveae*, and we hypothesized that *WRR4B* probably also contributes to the disease resistance against other PM pathogens. To test this hypothesis, we isolated the PMs from the hosts of *Vigna unguiculata*, *Murraya exotica* and *Solanum lycopersicum* and individually identified them as *Podosphaera xanthii*, *Erysiphe quercicola* and *Erysiphe neolycopersici* through the morphological characteristics (Figure S1) and the rRNA internal transcribed spacer (ITS) sequencing analysis (Figure S2). WT Col‐0 and *wrr4b‐1* mutants were inoculated with PM isolates *P. xanthii* JD 2202, *E. quercicola* JLX 2201 and *E. neolycopersici* FQ 2205. *P. xanthii* JD 2202 is a non‐adapted PM on *Arabidopsis* that is capable of forming initial haustoria but is arrested before sporulation. Until 12 dpi, no obvious symptoms were observed both in the WT Col‐0 and *wrr4b‐1* mutants (Figure 6a). However, microscopy analysis showed that JD 2202 developed a few conidiospores on the *wrr4b‐1* mutant but not on WT Col‐0, which only exhibited sparse hyphal networks (Figure 6b,c). By 10 dpi of JLX 2201, compared with the chlorosis symptoms of Col‐0, the *wrr4b‐1* mutant displayed typical PM white patches (Figure 6a), and there were more conidiospores produced on the *wrr4b‐1* mutant than on Col‐0 (Figure 6b,c). Tomato PM has been reported as a well‐adapted PM on Col‐0 (Xiao et al., 2005; Zhang et al., 2018). We also saw some FQ 2205 fungal growth on Col‐0 at 6 dpi. However, the growth was more pronounced on *wrr4b‐1*, where we could see early PM symptoms, which were not present yet on Col‐0 leaves (Figure 6a). Furthermore, the whole *wrr4b‐1* mutant leaves were covered with dense hyphal networks and lots of conidiospores (Figure 6b,c). However, on Col‐0 leaves, dendritic hyphal networks with a few conidiospores were observed and did not fuse together (Figure 6b,c). Overall, these results indicated that *WRR4B* contributes to a broad‐spectrum disease resistance against *P. xanthii*, *E. quercicola* and *E. neolycopersici*.

## 3 DISCUSSION

Natural rubber, an important raw industrial material, is primarily derived from plantations of rubber trees (*H. brasiliensis*); however, PM disease of rubber trees, caused by *O. heveae*, seriously affects yields of rubber latex globally. Almost all commercially cultivated rubber trees are prone to infection by this disease because they have a very narrow genetic background cloned from limited germplasm resources. A whole‐genome sequencing analysis of *H. brasiliensis* revealed that typical *NLR*, *EDS1*‐like and *PAD4*‐like genes are present in rubber trees (Tang et al., 2016), indicating that the ETI pathway functions in this plant species. It is therefore vital to discover resistance genes and breed resistant cultivars to combat PM disease in rubber trees. A previous study showed that *O. heveae*‐triggered disease resistance responses in WT *Arabidopsis* (Mei et al., 2016). In the present study, a *TNL* gene, *WRR4B*, was found to confer resistance against *O. heveae* in WT *Arabidopsis*, which may enable the generation of resistant rubber trees by transforming them with the *Arabidopsis WRR4B* gene.

The prototypical *R* genes responsible for resistance against PM fungi have not been found in *Arabidopsis*. The ecotype Ms‐0 is a resistant accession in which the PM‐resistance gene *RESISTANCE TO POWDERY MILDEW 8* (*RPW8*) was first characterized (Xiao et al., 2001). *RPW8* is not a typical *NLR* gene; it encodes a predicted N‐terminal transmembrane domain, a central CC domain and C‐terminal repeats (Barragan et al., 2019; Zhong & Cheng, 2016). In the present study, the loss of *WRR4B* resulted in significantly higher host cell entry ratios and fungal growth, as well as supporting the formation of conidiospores and mature conidia during *O. heveae* infection, which indicated that *WRR4B* is involved in both penetration and post‐penetration resistance against *O. heveae*. However, the susceptible phenotypes of *wrr4b* did not reach the level of the *eds1* mutant, especially the appearance of serious necrosis symptoms, apparent cell death and H_2_O_2_ production developed on *wrr4b* mutants at the later stage of infection, suggesting that *WRR4B* partially contributed to the disease resistance against *O. heveae* and that other *R* gene(s) in response to *O. heveae* should still be present on Col‐0. In this case, we also examined whether *WRR4A*, a homologue of *WRR4B*, is required for resistance against *O. heveae*. No obvious white patches were observed on *wrr4a* mutants. By 15 dpi, both WT and *wrr4a* mutants exhibited typical leaf chlorosis symptoms (Figure S3). But with the microscopy assay, the hyphal networks developed by *O. heveae* HN1106 on *wrr4a* mutants were slightly denser than on WT Col‐0 (Figure S3), suggesting that *WRR4A* may also partially contribute to the resistance against *O. heveae*. Nonetheless, it remains unclear whether there is a functional redundancy between WRR4A and WRR4B in the resistance against *O. heveae*.

In unchallenged healthy plants, *R* genes are generally expressed at a low level (Lai & Eulgem, 2018; Tan et al., 2007). Several *R* genes have been found to be induced by different pathogens; for example, sugar beet (*Beta vulgaris*) *Heterodera schachtii* (*Hs1* ^*pro‐1*^) (Cai et al., 1997), barley (*Hordeum vulgare*) *Mildew resistance locus a* (*Mla*) (Halterman et al., 2003) and rice (*Oryza sativa*) *Xa1* (Yoshimura et al., 1998) and *Xa21* (Gu et al., 2005). In this study, RNA‐seq analysis showed that *WRR4B* was downregulated, not induced, at 2 dpi with *O. heveae*, which appears to be inconsistent with the above notion. Nevertheless, the further RT‐qPCR analysis of earlier time points after *O. heveae* infection displayed that the *WRR4B* gene was induced by 12 hpi and 24 hpi and later decreased by 48 hpi. Therefore, the expression pattern of *WRR4B* upon *O. heveae* infection was upregulated at first and then downregulated to a lower level. The *WRR4A* gene also displayed a similar expression pattern (Figure S4). It is probably implemented by effector protein(s) secreted from *O. heveae* to minimize the pathogen recognition by the immune surveillance system. EDS1, PAD4, SARD1 and CBP60g are required for the upregulation of SA and NHP biosynthesis genes such as *ICS1* and *FMO1* (Ngou et al., 2022; Zeier, 2021), which comprise a positive feedback loop to regulate the signalling pathway. The activation of the TNLs upregulates the expression of these genes and promotes downstream immunity and cell death (Ngou et al., 2022; Zeier, 2021). Here, we explored whether these components are regulated by *WRR4B*, revealing that the *O. heveae‐*induced *EDS1*, *PAD4*, *SARD1, CBP60g, ICS1* and *PR1* expression levels were significantly lower in the *wrr4b* mutant than the WT at 2 dpi, but not at 4 and 8 dpi. This indicates that *WRR4B* takes part in the immune signalling positive feedback loop involving SA production to regulate resistance against *O. heveae*. In addition, *WRR4B* can also trigger self‐amplification, which is consistent with previous reports that a positive amplification loop exists in TIR domain signalling (Roberts et al., 2013). Overall, these results suggest that WRR4B, being a TNL, has NADase activity that makes AMP, ADP and ATP‐related products, which activate EDS1 complexes.

*WRR4B* was first characterized as an *R* gene against the white rust pathogen *A. candida* (Cevik et al., 2019). Four different CCG effector proteins in Ac2V have been found to be recognized by *WRR4B*, triggering the HR in a *WRR4B*‐dependent manner in *Arabidopsis* and *N. benthamiana* (Redkar et al., 2023). The genome and transcriptome of *O. heveae* strain HO‐73 have been sequenced, revealing that 133 candidate effector proteins are present in this strain.

Like *O. heveae* HN‐1106, *O. heveae* HO‐73 also mediated a more susceptible phenotype in the *wrr4b* mutant (Figure S5), indicating that the effector proteins corresponding to *WRR4B* should be present in *O. heveae* HO‐73. However, amino acid BLAST searches revealed that none of these 133 effectors display a high similarity to the four CCG effector proteins of Ac2V, suggesting that the effector proteins of *O. heveae* recognized by *WRR4B* are probably different from those of *A. candida*. In addition, *WRR4B* contributes to a broad‐spectrum disease resistance against *P. xanthii*, *O. heveae*, *E. quercicola* and *E. neolycopersici*, indicating that there may exist one or more conserved effector protein(s) recognized by *WRR4B* that are differentially distributed in these four PMs.

It has been shown that many NLRs function with helper NLRs, also termed RNLs due to their N‐terminal RPW8‐like CC domains, to transduce immune signals (Castel et al., 2019; Chai et al., 2023; Saile et al., 2020). In *Arabidopsis*, there are five active RNLs: activated disease resistance 1 (ADR1), ADR1‐like 1 (ADR1‐L1), ADR1‐L2, N requirement gene 1.1 (NRG1.1) and NRG1.2, which are partially redundant regulators of immunity and cell death downstream of TNLs (Castel et al., 2019; Saile et al., 2020; Wu et al., 2019). Interestingly, a newly defined TNL, the suppressor of ADR1‐L2 1 (SADR1), has been found to be required for the phenotypes driven by ADR1‐L2 autoactivity (Jacob et al., 2023), suggesting that TNLs can function downstream of RNL activation. In this scenario, given that *WRR4B* partially contributes to the disease resistance against *O. heveae* and mediates a broad‐spectrum resistance against many PM species and white rust pathogens, it is postulated that WRR4B may also act as a helper and together with other functional redundant regulators to fine‐tune the signalling pathways. In addition, although *WRR4B* confers race‐specific resistance to *A. candida*, virulent races of *Albugo* might have a strategy to overcome the *WRR4B*‐mediated resistance rather than lacking cognate avirulence genes.

## 4 EXPERIMENTAL PROCEDURES

### 4.1 Plant materials

The *Arabidopsis* ecotype Col‐0 was used as the WT in this study. The *Arabidopsis eds1‐2* mutant was previously developed by Aarts et al. (1998) and was backcrossed multiple times into the Col‐0 background. The T‐DNA insertion lines SALK_053459, SALK_104727C, SALK_032836C, SALK_139476, CS854738, SALK_047364, SALK_029707, SALK_084173C, SALK_127114, SALK_061751C, SALK_040895C, SALK_072335C, SALK_013292, SALK_148037C and SALK_133759 were obtained from http://arabidopsis.info. *nb‐eds1* mutant (Gantner et al., 2019).

### 4.2 Powdery mildew infections

The *O. heveae* strain HN1106 used in this study was maintained in the leaves of the susceptible *H. brasiliensis* clone 73397; *P. xanthii* JD 2202 was maintained in the leaves of *V. unguiculata* ‘ZhiJiang 19’; *E. quercicola* JLX 2201 was maintained in the leaves of *M. exotica*; *E. neolycopersici* FQ 2205 was maintained in the leaves of *S. lycopersicum* ‘YaShu 6’. Mature PM conidia were used as a source of inoculum. Actively growing fungal spores were dusted on top of the modified settling tower (diameter, 40 cm; height, 60 cm) and covered with a nylon mesh, after which the plants were placed under the settling tower for 1 h to be inoculated before being moved to the growth chamber.

### 4.3 Host cell entry assay

Three *O. heveae*‐infected leaves were harvested at 1 dpi and stained with Coomassie brilliant blue. The numbers of germinated fungal sporelings were counted, and the proportion of sporelings that developed secondary and more than secondary hyphae in all the germinated sporelings was assessed (a minimum of 50 germinated sporelings/leaves evaluated). The fungal penetration ratio of each plant was quantified in at least three independent experiments.

### 4.4 Analysis of fungal growth

Three independent plants per line were inoculated with a low density of *O. heveae*, and eight leaves per plant were harvested at 2 dpi and stained with Coomassie brilliant blue. At least 20 images of single colonies per line were taken and analysed using MIE v. 3.1 software (http://www.miesoftware.com/).

### 4.5 Quantification of conidiospores per colony

Eight inoculated *Arabidopsis* leaves were harvested at the indicated time and stained with Coomassie brilliant blue. The conidiospores produced by each of at least 20 colonies were counted. This procedure was repeated two to four times.

### 4.6 RNA isolation and RT‐qPCR

Five‐week‐old *Arabidopsis* leaves were inoculated with *O. heveae*, and their total RNA was isolated at the indicated time points. A 3–5 μg aliquot of RNA was used for the cDNA synthesis. The mRNA abundance was quantified using RT‐qPCR with a SYBR Premix Ex Taq Kit (Takara Bio). *Arabidopsis ACTIN* was used as the reference gene. Primers 5′‐TGGTGGAAGCACAGAAGTTG‐3′ and 5′‐GATCCATGTTTGGCTCCTTC‐3′ were used to amplify *ACTIN*; primers 5′‐TGGAGATTTCGGGATTGCTTTCA‐3′ and 5′‐GCTCCATCATAATCTAAATCAAG‐3′ were used to amplify *WRR4B*; primers 5′‐TGCAAAGAATATATAGGACAAAT‐3′ and 5′‐AGTCGAAACATCTCTTGCTATCT‐3′ were used to amplify *WRR4A*; primers 5′‐CGAAGACACAGGGCCGTA‐3′ and 5′‐AAGCATGATCCGCACTCG‐3′ were used to amplify *EDS1*; primers 5′‐GGTTCTGTTCGTCTGATGTTT‐3′ and 5′‐GTTCCTCGGTGTTTTGAGTT‐3′ were used to amplify *PAD4*; primers 5′‐TTCTGGGCTCAAACACTAAAAC‐3′ and 5′‐GGCGTCTTGAAATCTCCATC‐3′ were used to amplify *ICS1*; primers 5′‐TCAAGGCGTTGTGGTTTGTG‐3′ and 5′‐CGTCAACGACGGATAGTTTC‐3′ were used to amplify *SARD1*; primers 5′‐GATGACATGACCTCAAGCTG‐3′ and 5′‐TTAACCTTACACCACCTGGC‐3′were used to amplify *CBP60g*; and primers 5′‐TACGCAGAACAACTAAGAGG‐3′ and 5′‐TCGTTCACATAATTCCCACG‐3′ were used to amplify *PR1*. The RT‐qPCR conditions were as follows: 95°C for 2 min, followed by 40 cycles of 95°C for 15 s, 55°C for 15 s and 72°C for 25 s. The expression levels were normalized to those of the *ACTIN* control, and relative expression values were determined against uninfected samples or wild‐type Col‐0 using the comparative *C*_t_ method.

### 4.7 Trypan blue staining

Five‐week‐old *Arabidopsis* plant leaves were inoculated with *O. heveae*, and leaves were harvested at 6 and 12 dpi. Fungal structures and dead plant cells were stained with trypan blue and cleared with chloral hydrate overnight at room temperature (Frye & Innes, 1998). The cleared leaves were mounted under coverslips in 50% glycerol and examined under a microscope.

### 4.8 H_2_O_2_ production assay

Five‐week‐old *Arabidopsis* plant leaves were inoculated with *O. heveae* HN1106, and leaves were collected at 12 dpi. H_2_O_2_ was detected by staining them with 3,3′‐diaminobenzidine‐HCl (pH 3.8) for 8 h, followed by clearing in 95% ethanol overnight at room temperature (Xiao et al., 2003). The cleared leaves were mounted under coverslips in 50% glycerol and examined under a microscope.

### 4.9 Coomassie brilliant blue staining

Inoculated leaves were harvested and cleared in an ethanol solution (containing 6.7% phenol, 6.7% lactic acid, 13.3% glycerol, 6.7% water) 8 mL overnight. Fungal structures in the cleared leaves were stained with an ethanolic solution containing 0.6% Coomassie brilliant blue for 30 s and immediately washed by water (Lipka et al., 2005). The cleared leaves were mounted under coverslips in 50% glycerol and examined under a microscope.

### 4.10 Immunoblot analysis

*Arabidopsis* proteins were extracted with a buffer containing 50 mM Tris–HCl (pH 7.5), 150 mM NaCl, 5 mM EDTA, 0.1% Triton X‐100, 1 mM dithiothreitol, 2 mM NaF and protease inhibitor cocktail (Roche). The protein samples were electrophoresed through a 10% SDS‐PAGE gel, and the protein was electrotransferred onto an Immobilon P membrane (Millipore Sigma). Immunodetection was performed using a 1:5000 dilution of an anti‐FLAG monoclonal antibody (Millipore Sigma). The blot was then hybridized with horseradish peroxidase‐conjugated secondary antibodies and visualized with Amersham ECL Western Blotting Detection Reagents (Cytiva).

### 4.11 Transient expression in *N. benthamiana*

The full‐length *WRR4B* sequence was PCR‐amplified from *Arabidopsis* Col‐0 genomic DNA using the following primers: 5′‐GTCGACATGGCTTCTTCTTCTTCTTC‐3′ and 5′‐TTCGAAGAAAAGATCAAAGTAGATGG‐3′. The reverse primer 5′‐TTCGAAAGCGATGTTGCCCACATAGGTC‐3′ was used to amplify the *WRR4B* TIR domain fragment. The fragments were ligated into pER8, which had been digested with XhoI and BstBI. Overnight bacterial cultures of *A. tumefaciens* GV3101 were harvested using centrifugation. The cells were resuspended in induction buffer (10 mM MES pH 5.6, 10 mM MgCl_2_, 150 μM acetosyringone) to an OD_600_ of 0.8 and incubated for 2 h at room temperature. The agrobacteria were then infiltrated into 5‐week‐old *N. benthamiana* leaves using a 1‐mL needleless syringe. To induce the expression of the transgene, the plants were sprayed with 50 μM oestradiol containing 0.01% Silwet L‐77 at 1 dpi.

## Supporting information


**Figure S1.** The isolation and identification of powdery mildews (PMs) *Podosphaera xanthii*, *Erysiphe quercicola* and *Erysiphe neolycopersici*. (a) The PM symptoms on *Vigna unguiculata*, *Murraya exotica* and *Solanum lycopersicum* leaves. (b) The observation of the mycelial and conidiospores morphology of the isolated species *P. xanthii* JD 2202, *E. quercicola* JXL 2201 and *E. neolycopersici* FQ 2205. Bar, 30 μm. These experiments were repeated twice with similar results.

**Figure S2.** Construction of phylogenetic trees of three powdery mildews, *Podosphaera xanthii* JD 2202, *Erysiphe quercicola* JXL 2201 and *Erysiphe neolycopersici* FQ 2205, based on rRNA internal transcribed spacer (ITS) sequences.

**Figure S3.** Symptoms and light micrographs of *Arabidopsis* Col‐0 and *wrr4a* mutants infected with *Oidium heveae* HN1106 at 15 days post‐inoculation (dpi). Five‐week‐old *Arabidopsis* wild‐type (WT) Col‐0 and *wrr4a* mutants were inoculated with *O. heveae* HN1106. (a) Symptoms were photographed at 15 dpi. (b) Light microscopy images were taken after fungal structures had been stained with Coomassie brilliant blue at 15 dpi. Bar, 100 μm. These experiments were repeated twice with similar results.

**Figure S4.** The expression analysis of *WRR4A* in *Arabidopsis* wild‐type (WT) Col‐0 and *wrr4b* mutants. Five‐week‐old *Arabidopsis* wild‐type (WT) and *wrr4b* mutants were inoculated with *Oidium heveae* HN1106. The abundance of *WRR4A* mRNA was determined at the indicated time points after *O. heveae* inoculation using reverse transcription‐quantitative PCR. The data represent the mean ± *SD* of three independent experiments and six RNA replicates for each experiment. These experiments were repeated twice with similar results.

**Figure S5.** Symptoms and light micrographs of *Arabidopsis* Col‐0 wild‐type (WT) and *wrr4b‐1* mutants infected with *Oidium heveae* HO‐73 at 10 days post‐inoculation (dpi). Five‐week‐old *Arabidopsis* WT and *wrr4b‐1* mutants were inoculated with *O. heveae* HO‐73. (a) Symptoms were photographed at 10 dpi. (b) Light microscopy images were taken after fungal structures had been stained with trypan blue and Coomassie brilliant blue at 10 dpi. Bar, 200 μm. These experiments were repeated twice with similar results.

**Table S1.** Differentially expressed *TIR*‐*NBS*‐*LRR* genes at 2 days post‐inoculation (dpi) relative to 0 dpi.

**Table S2.** Infection phenotypes of *Arabidopsis* Col‐0 (wild‐type, WT) and T‐DNA insertion lines for 12 *TIR*‐*NB*‐*LRR* genes, following a challenge by *Oidium heveae* HN1106.

## Data Availability

The authors confirm that the data supporting the findings of this study are available within the article and its supplementary materials.
