# Supplementary figures and images for: WRR4B contributes to a broad‐spectrum disease resistance against powdery mildew in Arabidopsis
Source: Mol Plant Pathol. 2024 Jan 8;25(1):e13415. doi: 10.1111/mpp.13415 (PMC10777751; doi:10.1111/mpp.13415)

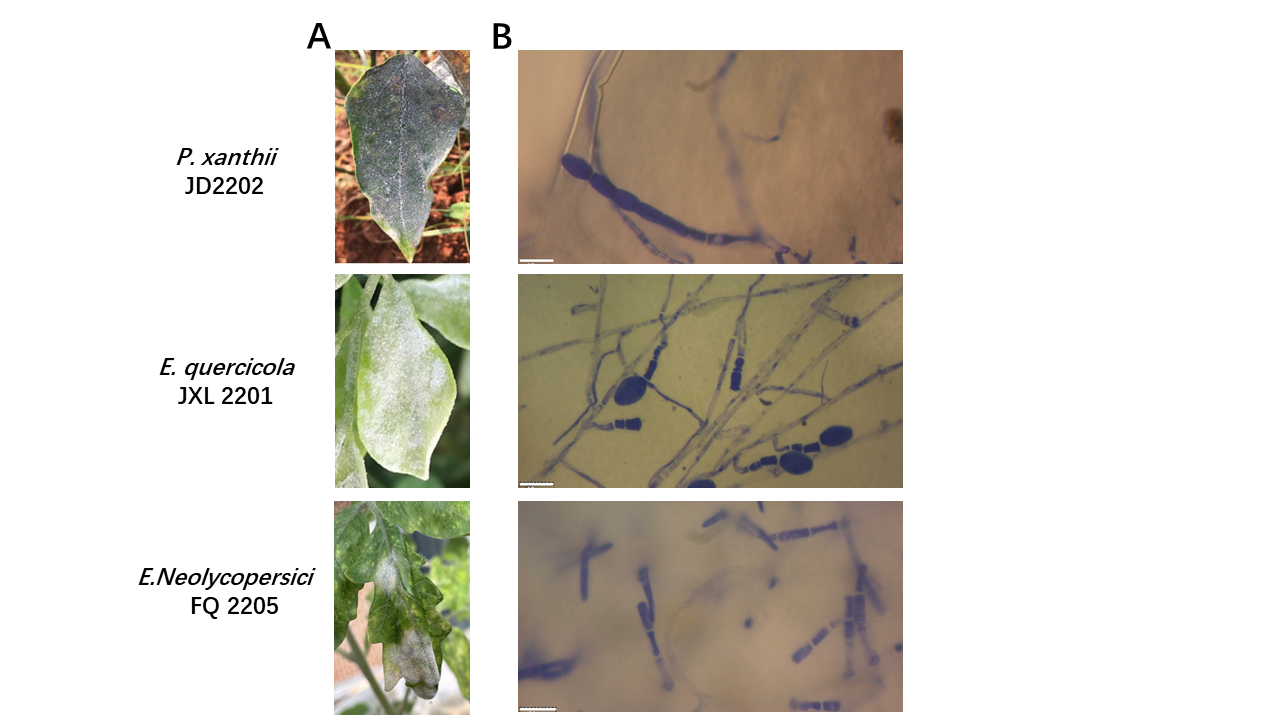

Supplement: Supplementary file 1 — Figure S1. The isolation and identification of powdery mildews (PMs) Podosphaera xanthii, Erysiphe quercicola and Erysiphe neolycopersici. (a) The PM symptoms on Vigna unguiculata, Murraya exotica and Solanum lycopersicum leaves. (b) The observation of the mycelial and conidiospores morphology of the isolated species P. xanthii JD 2202, E. quercicola JXL 2201 and E. neolycopersici FQ 2205. Bar, 30 μm. These experiments were repeated twice with similar results. [file MPP-25-e13415-s003.tif]

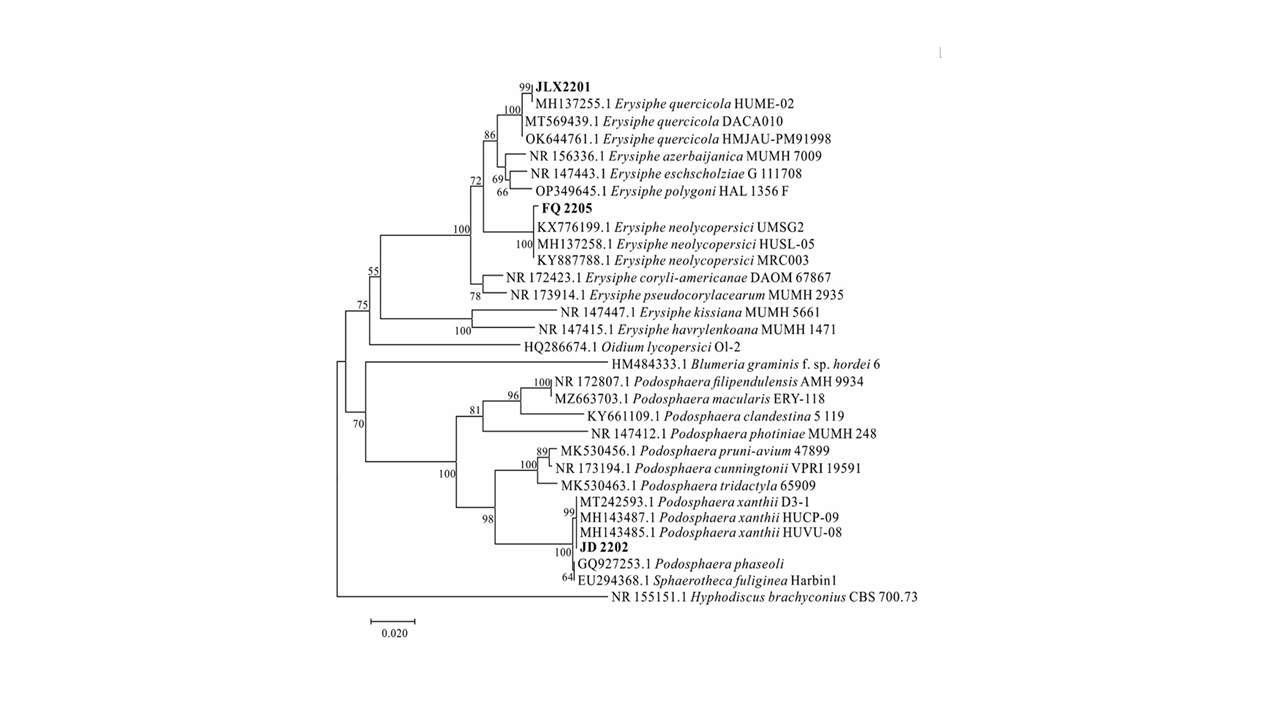

Supplement: Supplementary file 2 — Figure S2. Construction of phylogenetic trees of three powdery mildews, Podosphaera xanthii JD 2202, Erysiphe quercicola JXL 2201 and Erysiphe neolycopersici FQ 2205, based on rRNA internal transcribed spacer (ITS) sequences. [file MPP-25-e13415-s006.tif]

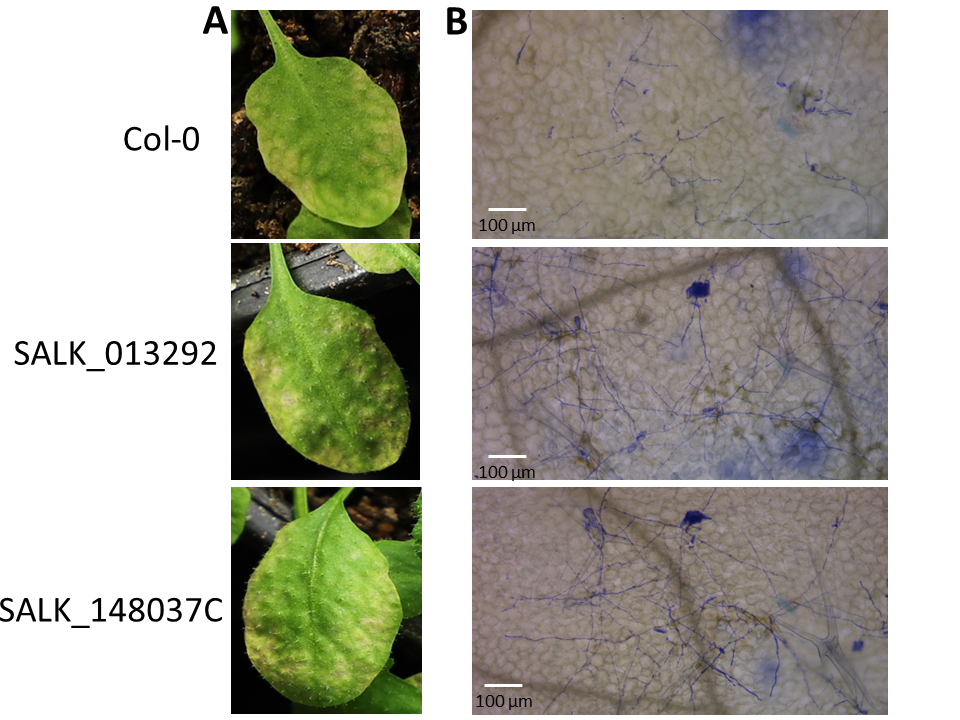

Supplement: Supplementary file 3 — Figure S3. Symptoms and light micrographs of Arabidopsis Col‐0 and wrr4a mutants infected with Oidium heveae HN1106 at 15 days post‐inoculation (dpi). Five‐week‐old Arabidopsis wild‐type (WT) Col‐0 and wrr4a mutants were inoculated with O. heveae HN1106. (a) Symptoms were photographed at 15 dpi. (b) Light microscopy images were taken after fungal structures had been stained with Coomassie brilliant blue at 15 dpi. Bar, 100 μm. These experiments were repeated twice with similar results. [file MPP-25-e13415-s004.tif]

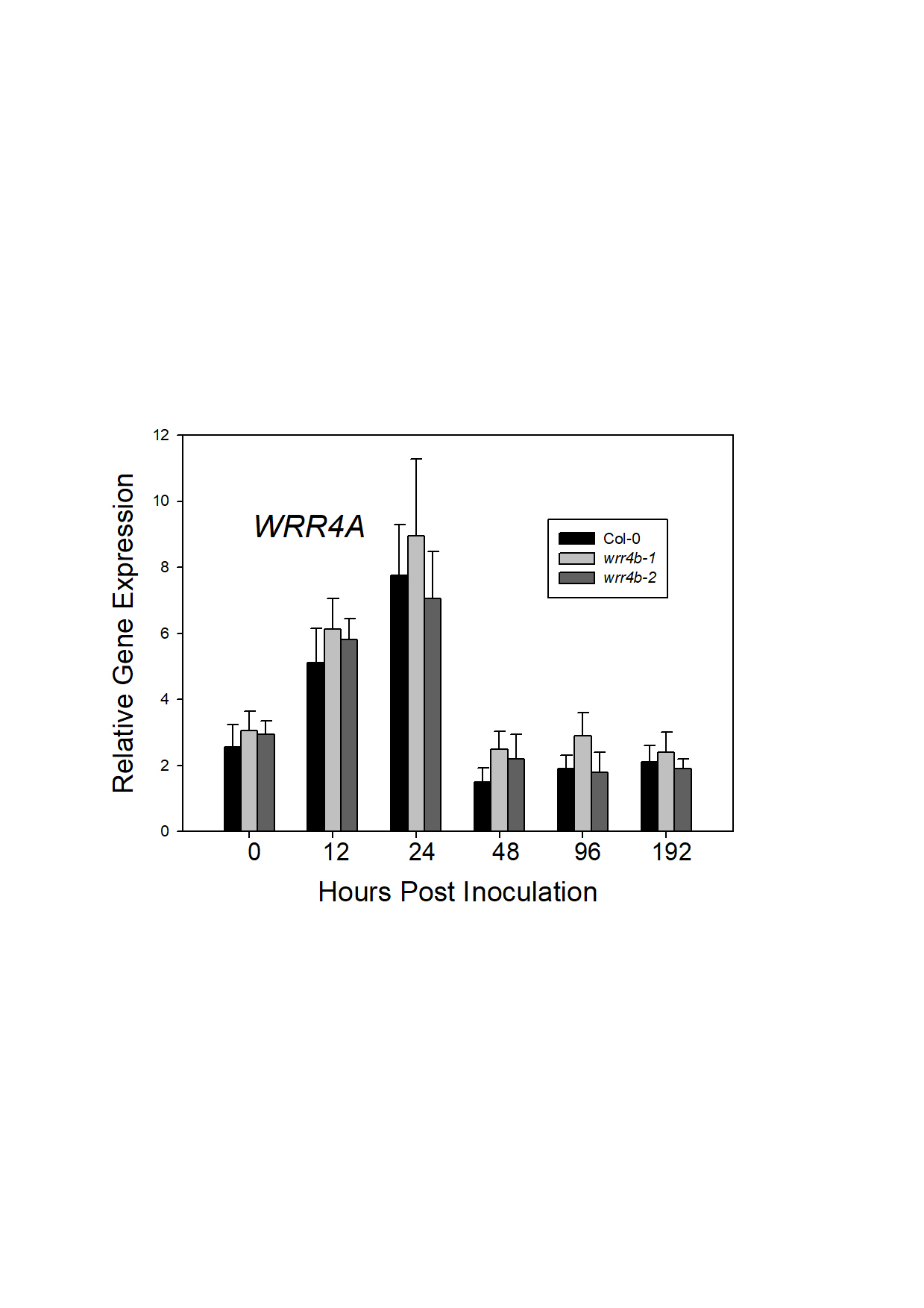

Supplement: Supplementary file 4 — Figure S4. The expression analysis of WRR4A in Arabidopsis wild‐type (WT) Col‐0 and wrr4b mutants. Five‐week‐old Arabidopsis wild‐type (WT) and wrr4b mutants were inoculated with Oidium heveae HN1106. The abundance of WRR4A mRNA was determined at the indicated time points after O. heveae inoculation using reverse transcription‐quantitative PCR. The data represent the mean ± SD of three independent experiments and six RNA replicates for each experiment. These experiments were repeated twice with similar results. [file MPP-25-e13415-s007.JPG]

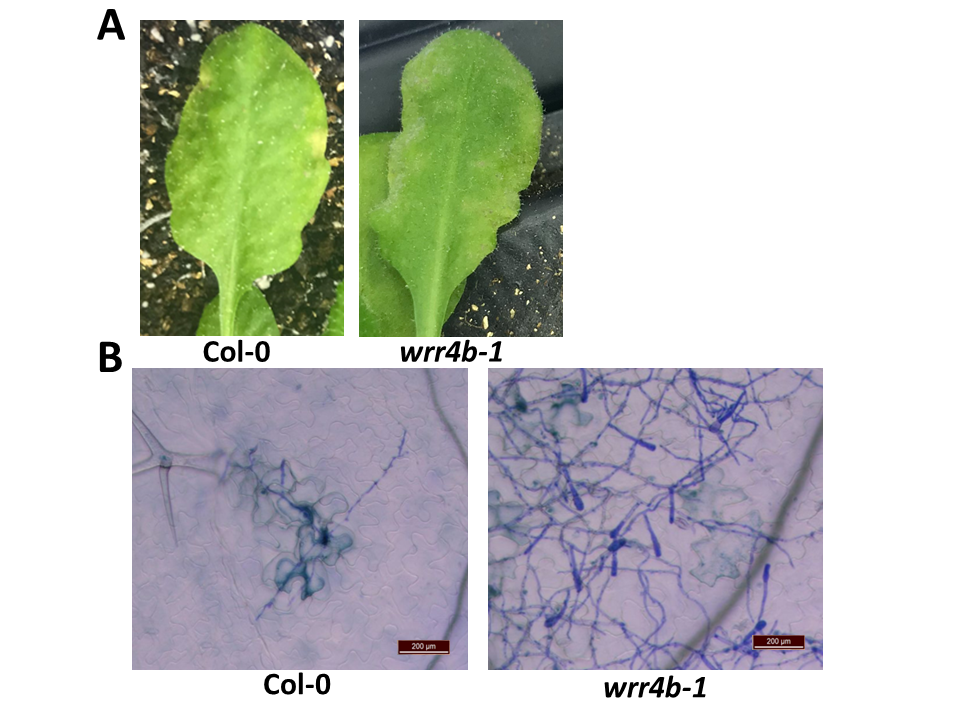

Supplement: Supplementary file 5 — Figure S5. Symptoms and light micrographs of Arabidopsis Col‐0 wild‐type (WT) and wrr4b‐1 mutants infected with Oidium heveae HO‐73 at 10 days post‐inoculation (dpi). Five‐week‐old Arabidopsis WT and wrr4b‐1 mutants were inoculated with O. heveae HO‐73. (a) Symptoms were photographed at 10 dpi. (b) Light microscopy images were taken after fungal structures had been stained with trypan blue and Coomassie brilliant blue at 10 dpi. Bar, 200 μm. These experiments were repeated twice with similar results. [file MPP-25-e13415-s005.tif]
