# Supplementary material for: WRR4B contributes to a broad‐spectrum disease resistance against powdery mildew in Arabidopsis
Source: Mol Plant Pathol. 2024 Jan 8;25(1):e13415. doi: 10.1111/mpp.13415 (PMC10777751; doi:10.1111/mpp.13415)
Supplement: Supplementary file 6 — Table S1. Differentially expressed TIR‐NBS‐LRR genes at 2 days post‐inoculation (dpi) relative to 0 dpi. [file MPP-25-e13415-s002.docx]

**Table S1** Differentially expressed *TIR*-*NBS*-*LRR* genes at 2 dpi relative to 0 dpi

| Gene ID | Fold change relative to 0 dpi* | T-DNA insertion mutant |
| --- | --- | --- |
| AT4G11340 | 6.10 | SALK_053459 |
| AT5G38350 | 5.16 | SALK_104727C |
| AT3G25510 | 1.83 | SALK_032836C |
| AT5G45000 | 1.69 | SALK_139476 |
| AT5G46260 | 1.47 | CS854738 |
| AT3G44480 | 1.44 | SALK_047364 |
| AT3G44670 | 1.42 | SALK_029707 |
| AT5G36930 | 1.30 | SALK_084173C |
| AT4G36150 | 1.20 | SALK_127114 |
| AT5G22690 | -1.72 | SALK_061751C |
| AT1G56540  (WRR4B) | -1.20 | SALK_040895C  SALK_072335C |
| AT1G63750 | -1.20 | SALK_133759 |

*log_2_ fold change (Col-0_2d/Col-0_0d)
