# Supplementary material for: WRR4B contributes to a broad‐spectrum disease resistance against powdery mildew in Arabidopsis
Source: Mol Plant Pathol. 2024 Jan 8;25(1):e13415. doi: 10.1111/mpp.13415 (PMC10777751; doi:10.1111/mpp.13415)
Supplement: Supplementary file 7 — Table S2. Infection phenotypes of Arabidopsis Col‐0 (wild‐type, WT) and T‐DNA insertion lines for 12 TIR‐NB‐LRR genes, following a challenge by Oidium heveae HN1106. [file MPP-25-e13415-s001.docx]

**Table S2** Infection phenotypes of Arabidopsis Col-0 (WT) and T-DNA insertion lines for 12 *TIR*–*NB*–*LRR* genes, following a challenge by *O. heveae* HN1106

| Genotype | White patches | Conidiospores, mature conidia | Chlorosis | Necrosis |
| --- | --- | --- | --- | --- |
| Col-0 | - | - | **+** | - |
| SALK_053459 | - | - | **+** | **-** |
| SALK_104727C | - | - | **+** | **-** |
| SALK_032836C | - | - | **+** | **-** |
| SALK_139476 | - | - | **+** | **-** |
| CS854738 | - | - | **+** | **-** |
| SALK_047364 | - | - | **+** | **-** |
| SALK_029707 | - | - | **+** | **-** |
| SALK_084173C | - | - | **+** | **-** |
| SALK_127114 | - | - | **+** | **-** |
| SALK_061751C | - | - | **+** | **-** |
| SALK_040895C  SALK_072335C  (WRR4B) | **+** | **+** | **-** | **+** |
| SALK_133759 | - | - | **+** | **-** |
